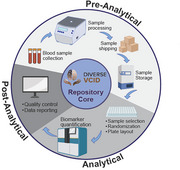# Harmonizing Biofluid Sample Collection and Biomarker Quantification in Diverse Vascular Contributions to Cognitive Impairment and Dementia (DVCID) Study

**DOI:** 10.1002/alz70856_099469

**Published:** 2025-12-24

**Authors:** Nopparat Suthprasertporn, Sitong Zhou, Jacob Conston, Ramses Denis‐Romero, Kelsey Erickson, Charles S. DeCarli, Myriam Fornage, Lee‐Way Jin

**Affiliations:** ^1^ University of California, Davis, Sacramento, CA, USA; ^2^ University of California Davis Medical Center, Sacramento, CA, USA; ^3^ Department of Neurology & Imaging of Dementia and Aging Laboratory, University of California, Davis, Davis, CA, USA; ^4^ The Brown Foundation Institute of Molecular Medicine, McGovern Medical School, The University of Texas Health Science Center at Houston, Houston, TX, USA; ^5^ University of California, Davis, Davis, CA, USA

## Abstract

**Background:**

The DVCID project, funded by the National Institute of Neurological Disorders and the National Institute on Aging, aims to enroll 2250 diverse at‐risk Americans from Black/African, Latino/Hispanic, and non‐Hispanic White backgrounds. The study integrates cognitive assessments, blood‐based analyses, and neuroimaging. As the Repository Core (RC) of the coordinating center at the University of California, Davis (UCD), our responsibilities include blood processing, banking, biomarker measurements, and analysis. Standard operating procedures (SOPs) are essential to ensure consistency, reliability, and comparability of the analytical results across the 20 participating sites.

**Method:**

We detailed the comprehensive strategies implemented by the RC to harmonize biofluid sample collection, processing, storage, and data management across sites. Pre‐analytical, analytical, and post‐analytical factors influencing sample integrity and data reliability were evaluated. Specifically, post‐analytical quality control included assessments of within‐run, between‐run, and between‐lot variabilities. Measurements were implemented to maintain consistent and reliable quantitative biomarker data, analyzed using Quanterix Simoa HD‐X™ Analyzer.

**Result:**

Harmonization strategies included standardized blood processing protocols, such as the use of approved vacutainer tubes, uniform blood handling steps, and consistent cryovial formats for storage. To minimize variability in biomarker quantification, we employed data harmonization approaches, including re‐analysis of a subset of samples for batch‐bridging purposes. Post‐analytical quality control measures like this ensured that within‐run, between‐run, and between‐lot variability remained within acceptable limits (CV ≤10‐15%). As a result, no significant variance in Quanterix biomarker data was observed across study sites, confirming that the implemented measures improved sample integrity and analytical consistency across sites.

**Conclusions:**

This work highlights the importance of harmonized strategies and SOPs for biofluid collection, processing, storage, and biomarker quantification in large, multi‐centered clinical research. The outlined practices ensure sample integrity and data consistency, enabling more reliable biomarker research. These methods also make cross‐cohort studies possible by improving data comparability. Such harmonization could facilitate larger‐scale investigations which advance the field of clinical and biomarker research.